# *SOX9* is a driver of aggressive prostate cancer by promoting invasion, cell fate and cytoskeleton alterations and epithelial to mesenchymal transition

**DOI:** 10.18632/oncotarget.24123

**Published:** 2018-01-10

**Authors:** Jeffrey C. Francis, Amy Capper, Jian Ning, Eleanor Knight, Johann de Bono, Amanda Swain

**Affiliations:** ^1^ Division of Cancer Biology, The Institute of Cancer Research, London SW3 6JB, UK; ^2^ Tumour Profiling Unit, The Institute of Cancer Research, London SW3 6JB, UK; ^3^ Division of Clinical Studies, The Institute of Cancer Research, London SM2 5NG, UK

**Keywords:** aggressive invasive prostate cancer, prostate cancer mouse models, cell lineage plasticity, epithelial to mesenchymal transition

## Abstract

Aggressive lethal prostate cancer is characterised by tumour invasion, metastasis and androgen resistance. Understanding the mechanisms by which localised disease progresses to advanced lethal stages is key to the development of effective therapies. Here we have identified a novel role for the transcription factor, SOX9, as a driver of aggressive invasive prostate cancer. Using genetically modified mouse models, we show that increased *Sox9* expression in the prostate epithelia of animals with *Pten* loss leads to a highly invasive phenotype and metastasis. In depth analysis of these mice and related *in vitro* models reveals that SOX9 acts a key regulator of various processes that together promote tumour progression. We show that this factor promotes cell lineage plasticity with cells acquiring properties of basal stem cells and an increase in proliferation. In addition, increased SOX9 leads to changes in cytoskeleton and adhesion, deposition of extracellular matrix and epithelia to mesenchyme transition, properties of highly invasive cells. Analysis of castrated mice showed that the invasive phenotype driven by SOX9 is independent of androgen levels. Our study has identified a novel driver of prostate cancer progression and highlighted the cellular and molecular processes that are regulated by *Sox9* to achieve invasive disease.

## INTRODUCTION

Prostate cancer is a leading cause of cancer mortality in men in the western world. Lethal aggressive disease is characterised by tumour invasion, metastasis and castration resistance [1]. Identifying and understanding the processes that drive prostate cancer progression is an important challenge. The *PTEN* (phosphatase and tensin homolog deleted on chromosome 10) tumour suppressor gene has been implicated in prostate cancer with late-stage disease showing loss of function in over 60% of samples [2, 3]. Mice lacking *Pten* in the prostate show prostate intraepithelial neoplasia (PIN) that progresses to adenocarcinoma in older animals with minimal invasive properties [4]. These animals have been used to identify factors that cooperate with *Pten* loss to drive prostate cancer progression to more aggressive stages [5, 6].

The transcription factor SOX9 has been shown to be a key regulator in various processes during embryogenesis, stem cell commitment and cancer. In the prostate, we and others have shown that it is expressed at early stages of organogenesis and required for prostate development [7, 8]. SOX9 has been implicated in prostate cancer, with high levels of SOX9 found in early stages of prostate neoplasia and high grade PIN in mice and associated with increasing Gleason grade in humans [9]. Proposed oncogenic mechanisms to achieve increased SOX9 include transcriptional activation by ERG [10], which is highly expressed in tumours with TMPRSS2:ERG fusions, and loss of *Zbtb7a*, encoding a protein that antagonizes SOX9 activity [11]. *in vivo* functional studies have shown that overexpression of *Sox9* in prostate epithelia in transgenic mice induced increased proliferation and high grade PIN in mice that also had a heterozygous *Pten* deletion [9]. Moreover, *Sox9* loss inhibited tumour formation in two prostate cancer mouse models (TRAMP and Myc overexpression)[7].

Here we identify a novel role for SOX9 at later stages of prostate cancer progression. Analyses of genetically modified mice show that overexpression of *Sox9* in the prostate of mice with a homozygous *Pten* deletion leads to highly invasive prostate cancer and metastasis. Our studies reveal that *Sox9* regulates various processes that together drive increased proliferation and concerted stromal cell invasion and metastasis, hallmarks of aggressive disease.

## RESULTS

### SOX9 promotes highly invasive tumour formation in *Pten* deficient prostates

**Figure 1:**
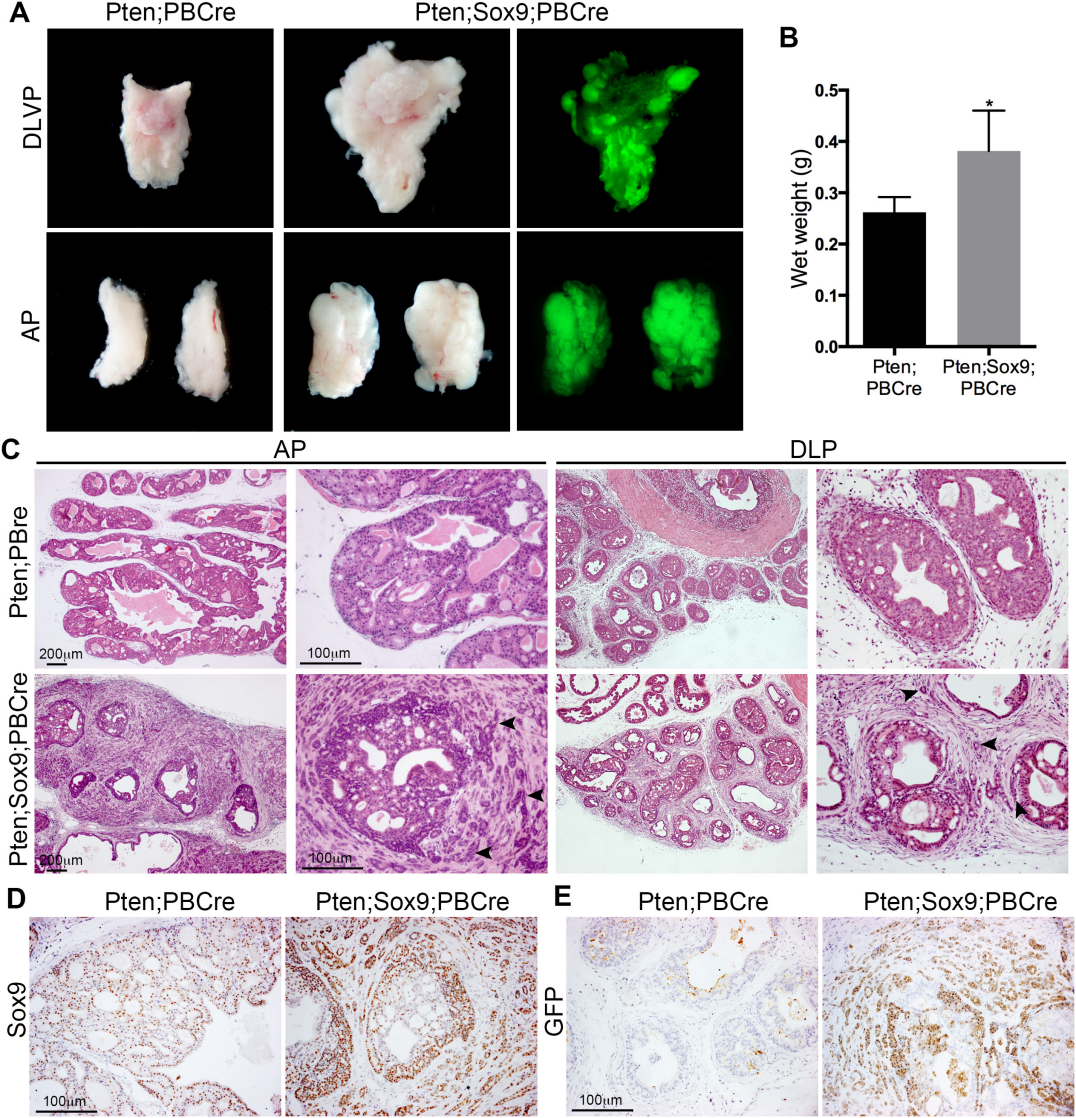
High levels of *Sox9* drive *Pten* loss neoplasia to highly invasive prostate cancer **(A)** Bright-field and GFP whole-mount mount images of prostates with *Pten* deletion (Pten;PBCre) or *Pten* deletion and *Sox9* overexpression (Pten;Sox9;PBCre). The dorsal-lateral-ventral lobes (DLVP) and anterior lobes (AP) from individual animals of each genotype are shown. **(B)** Wet weights of prostates with *Pten* deletion or *Pten* deletion and *Sox9* overexpression, ^*^ is p<0.05. **(C)** Haematoxylin and eosin (H&E) staining of sections of AP and dorsal-lateral lobes (DLP) of prostates with *Pten* deletion or *Pten* deletion and *Sox9* overexpression. Arrowheads indicate epithelial cells invading the stroma. **(D)** Sox9 staining and **(E)** GFP staining of sections of prostates with *Pten* deletion or *Pten* deletion and *Sox9* overexpression.

In an effort to determine the role of SOX9 in aggressive prostate cancer we generated genetically modified mice that express extra levels of *Sox9* in prostate epithelia in addition to a homozygous *Pten* deletion. For this, mice containing a construct where *Sox9* is conditionally expressed under the control of a ubiquitous promoter in tissues where Cre is expressed (Z/Sox9, [9]) were bred with mice with a homozygous *Pten* loxP containing allele (Pten^fl/fl^, [12]) and a transgene where Cre is driven by a Probasin derived promoter (PbCre4 [13]) (the Z/Sox9^tg/+^; *Pten*^fl/fl^; PbCre^tg/+^ mice are referred to as Sox9;Pten;PBCre mutants throughout the manuscript). This *Sox9* containing transgene also drives expression of GFP, allowing us to specifically follow prostate epithelial cells that overexpress *Sox9*. As expected, Sox9;Pten;PBCre mutant prostates were positive for GFP staining (Figure 1A). Initial analysis of prostates from 3 month old mice showed that Sox9;Pten;PBCre mutants had a larger size compared to Pten;PBCre mutants (Figure 1A and 1B). Sections of these prostates revealed prostatic ducts filled with cells and high grade PIN, as seen in Pten;PBCre mutant animals. In contrast to Pten;PBCre mutant animals, Sox9;Pten;PBCre prostates had abundant evidence of a highly invasive phenotype that was focal in nature and associated with an increase in stromal tissue (Figure 1C). The epithelial cells invading into the surrounding stroma expressed high levels of SOX9 (Figure 1D). As *Sox9* is expressed in normal prostate, we used GFP staining to reveal transgenic *Sox9* expression. This analysis confirmed that GFP positive cells show elevated levels of SOX9 and that the invading epithelial cells express transgenic *Sox9* (Figure 1E and Supplementary Figure 1 [9]). Consistent with the invasive phenotype we observe metastatic GFP positive cells in lymph nodes and lungs of 6 month old Sox9;Pten;PBCre mutants, a process that we do not observe in Pten;PBCre mutant animals at this stage (Supplementary Figure 2).

### Increased proliferation in Sox9;Pten;PBCre mutant prostates

**Figure 2:**
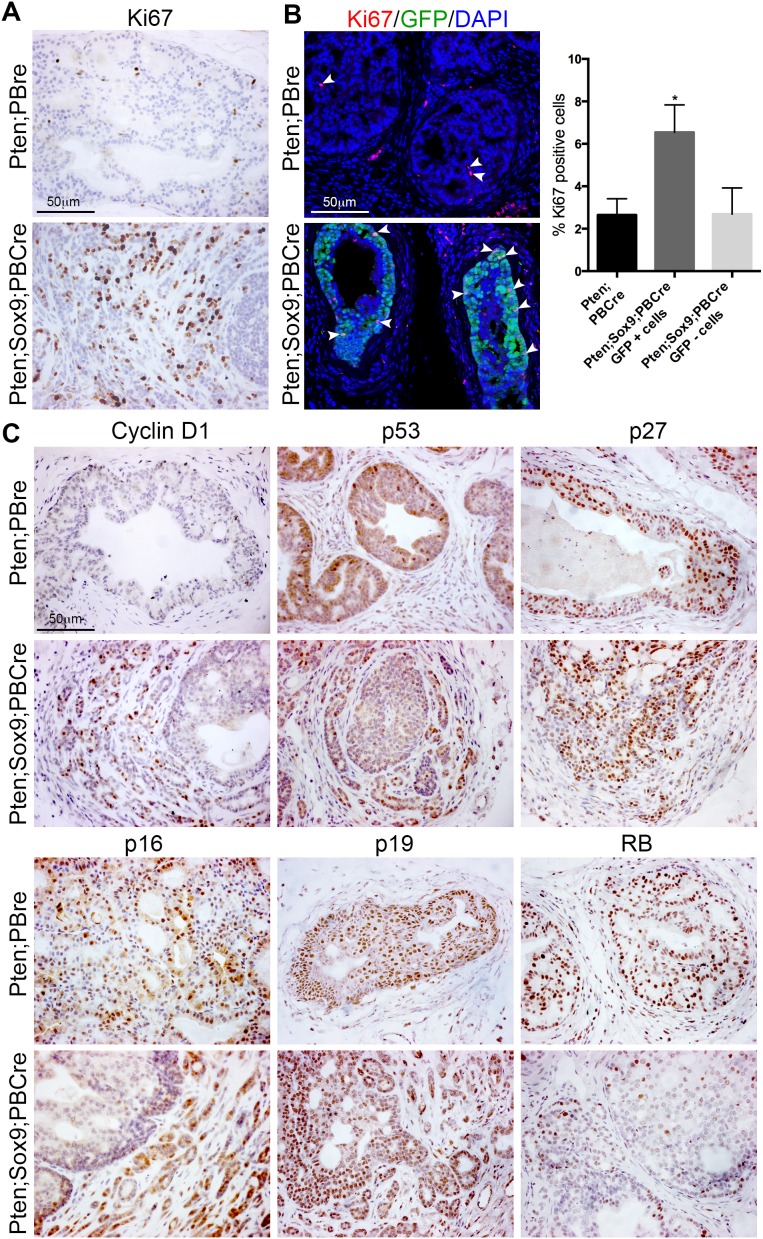
High levels of Sox9 promote proliferation of *Pten* deficient prostate cells **(A)** Ki67 staining of sections of prostates with *Pten* deletion or *Pten* deletion and *Sox9* overexpression. **(B)** Ki67 and GFP immunofluorescence staining of sections of prostates with *Pten* deletion or *Pten* deletion and *Sox9* overexpression. White arrowheads indicate Ki67 positive cells. Bar chart showing quantification of Ki67 positive cells in prostates with *Pten* deletion or *Pten* deletion and *Sox9* overexpression, ^*^ is p<0.05. For double mutant prostates, Ki67 positive cells were quantified from cells that overexpressed *Sox9* (GFP+) and cells that did not (GFP-). **(C)** Cyclin D1, p53, p27, p16, p19 and RB staining of sections of prostates with *Pten* deletion or *Pten* deletion and *Sox9* overexpression.

The larger size of Sox9;Pten;PBCre mutant prostates prompted us to look at proliferation in this tissue. To determine the specific effect of transgenic *Sox9* we stained prostate sections with Ki67, a marker of proliferation, and GFP antibodies. These studies showed that Sox9;Pten;PBCre mutant prostates have a significant increase in Ki67 positive epithelial cells that were also positive for GFP, compared to GFP negative cells or to Pten;PBCre mutant animals (Figure 2A and 2B). To understand the mechanisms relating to proliferation regulated by *Sox9*, we stained Sox9;Pten;PBCre mutant prostates with several markers and observed an increase in the expression of Cyclin D1, particularly in invasive regions (Figure 2C). Pten;PBCre mutant prostates have been reported to show senescence that is bypassed when other oncogenic insults such as *Tp53* or *Zbtb7a* loss are present [11, 14]. Due to the focal nature of the phenotype, we used antibody staining to investigate senescence associated genes and found no major difference in expression of p53, p27, p16 and p19 between Sox9;Pten;PBCre and Pten;PBCre mutant prostates (Figure 2C). Based on the study of mice with *Zbtb7a* and *Pten* loss, which show a decrease in PTEN induced senescence (PIS) and an increase in SOX9 transcriptional activity [11], we investigated the expression of the tumours suppressor retinoblastoma (RB). This analysis showed decreased RB expression in Sox9;Pten;PBCre mutant prostates compared to Pten;PBCre mutants (Figure 2C). In support of this, direct evidence that RB loss can overcome PIS has been shown in prostates that have both *Rb* and *Pten* deleted [15].

### Adhesion phenotype in Sox9;Pten;PBCre mutant prostates

**Figure 3:**
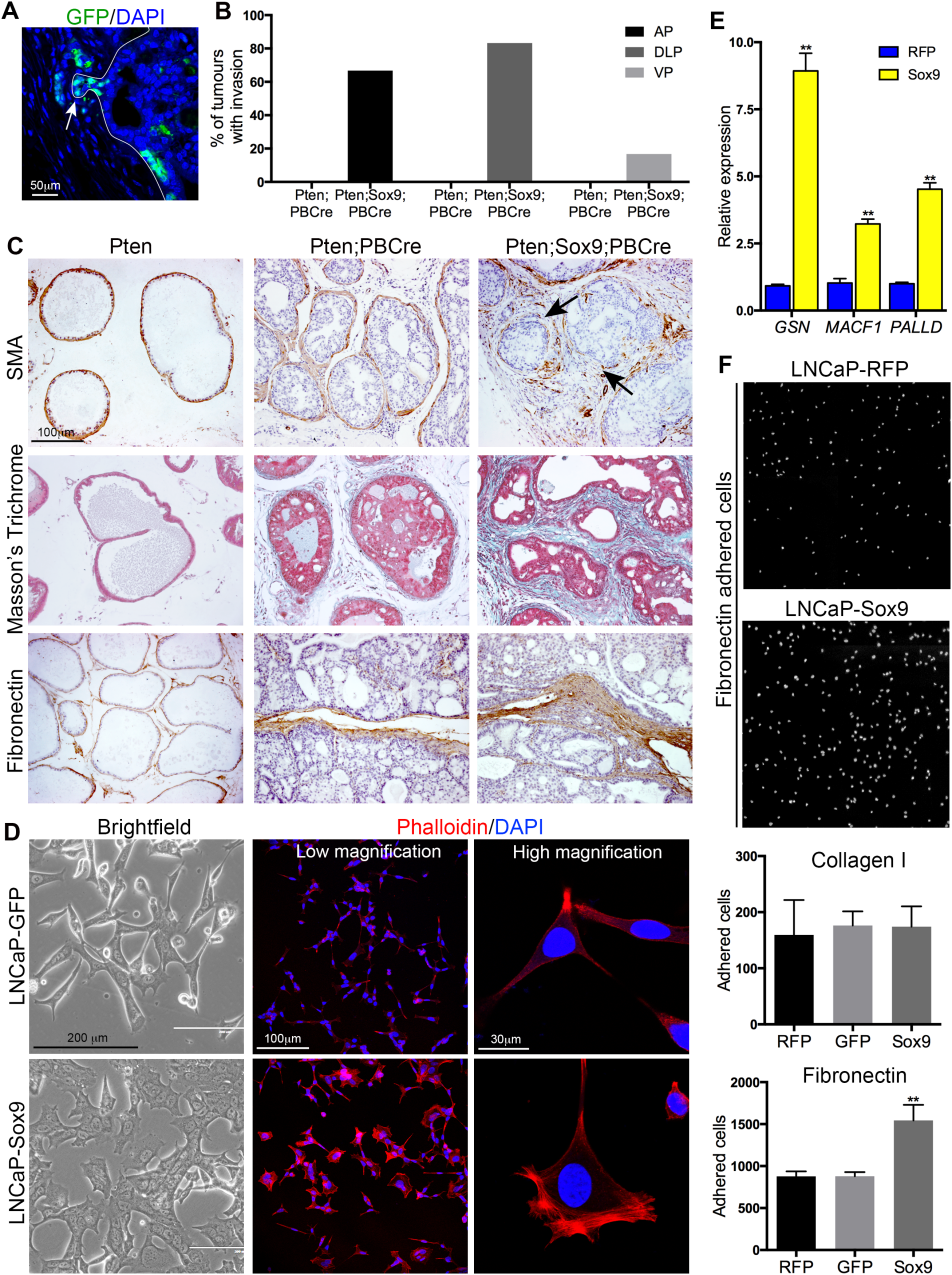
*Sox9* promotes epithelial invasion, F-actin remodelling and adhesion to Fibronectin **(A)** GFP immunofluorescence staining of a section of a prostate with *Pten* deletion and *Sox9* overexpression showing focal invasion of GFP positive cells. **(B)** Bar chart showing quantification of tumours with epithelial invasion in prostates with *Pten* deletion or *Pten* deletion and *Sox9* overexpression. AP is anterior lobes, DLP is dorsal-lateral lobes and VP is ventral lobes. **(C)** Smooth-muscle actin (SMA), Masson’s Trichrome, and Fibronectin staining of sections of *Pten* prostates, prostates with *Pten* deletion or prostates with *Pten* deletion and *Sox9* overexpression. Arrows mark areas that have lost SMA. **(D)** Bright-field images and Phalloidin staining with DAPI counterstain of LNCaP cells expressing GFP or Sox9. **(E)** Quantitative RT-PCR of *GSN*, *MACF1* and *PALLD* in LNCaP cells expressing RFP or Sox9. **(F)** Images of DAPI stained LNCaP cells expressing RFP or Sox9 adhered to Fibronectin. Bar chart of the number of LNCaP cells expressing RFP, GFP or Sox9 adhered to Collagen I or Fibronectin. ^**^ is p<0.01.

Further analysis of the invasive phenotype in Sox9;Pten;PBCre mutant prostates showed that it is focal and found in all prostatic lobes although a lower incidence is seen in the ventral prostate, possibly due to variation in the expression of Cre in different lobes (Figure 3A and 3B, [13]). It is characterised by the disruption of the stromal barrier, as shown by the interrupted smooth-muscle (SMA) actin staining pattern surrounding the duct (Figure 3C). An increase in stromal tissue is observed in the invasive regions as well as high levels of extra cellular matrix markers such as fibronectin and collagen when compared to Pten;PBCre mutant animals (Figure 2C). These studies indicated that high levels of *Sox9* lead to a change in adhesive properties. To investigate this hypothesis we overexpressed *SOX9* in LNCaP cells, which do not express PTEN. LNCaP cells with high levels of *SOX9* showed a difference in cellular morphology with a flatter appearance than control cells (Figure 3D). This difference was highlighted with Phalloidin staining, which showed an increase in F-actin in *SOX9* expressing cells (Figure 3D). Consistent with an effect of *Sox9* on adhesion, genes associated with adhesomes, *GSN*, *MACF1* and *PALLD*, were found to be increased in cells expressing high levels of *SOX9* (Figure 3E). Moreover, these cells showed increase adhesion to fibronectin but not to collagen (Figure 3F).

### Epithelia to mesenchyme transition in Sox9;Pten;PBCre mutant prostates

**Figure 4:**
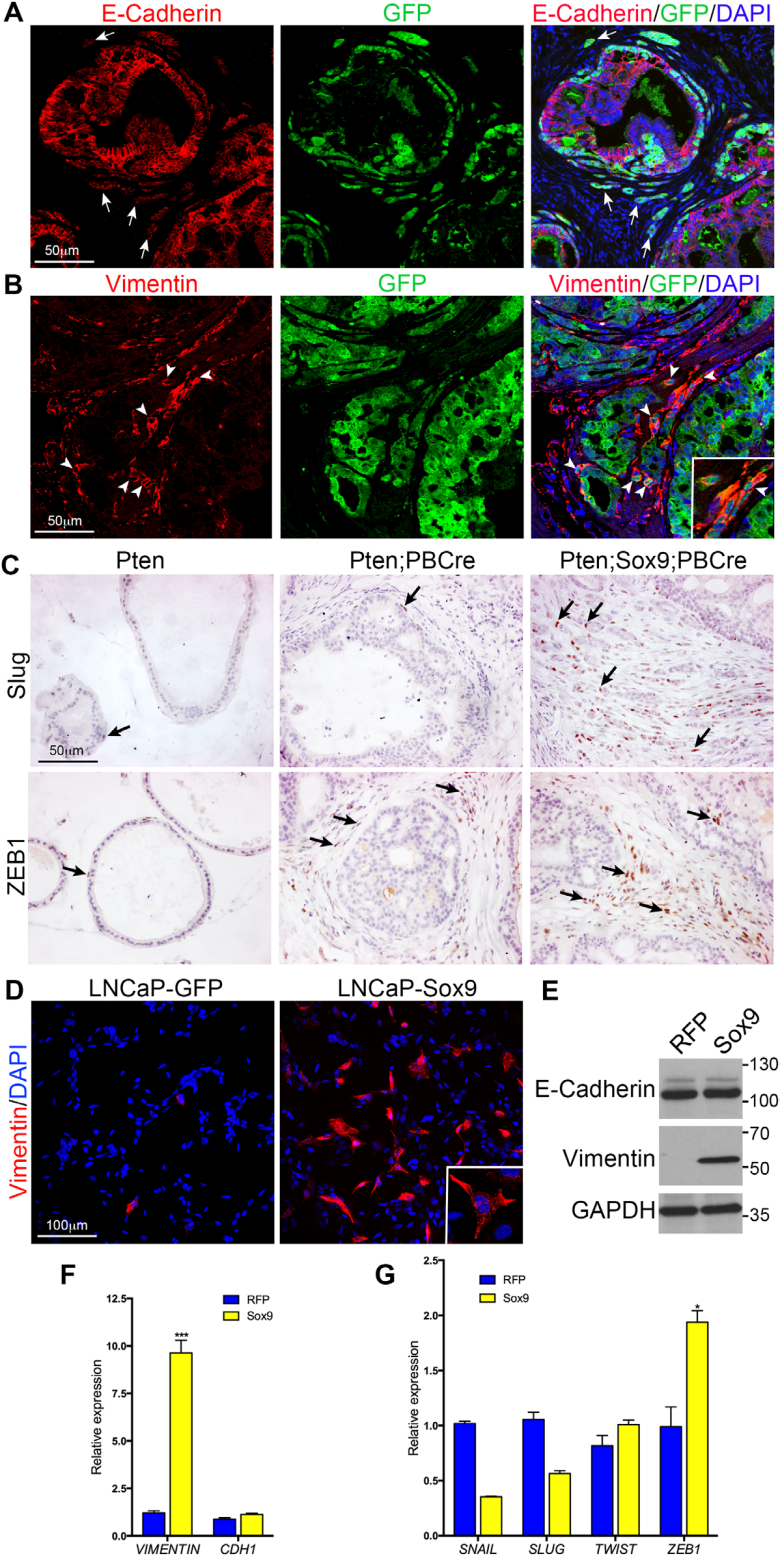
Sox9 promotes Epithelial to Mesenchymal Transition in *Pten* mutant prostate cells **(A)** E-Cadherin and GFP immunofluorescence staining of sections of prostates with *Pten* deletion and *Sox9* overexpression. White arrows mark invading cells that have reduced E-Cadherin expression. **(B)** Vimentin and GFP immunofluorescence staining of sections of prostates with *Pten* deletion and *Sox9* overexpression. White arrowheads mark invading cells that express Vimentin and GFP. **(C)** Slug and ZEB1 staining of sections of *Pten* prostates, prostates with *Pten* deletion or prostates with *Pten* deletion and *Sox9* overexpression. Arrows highlight cells with positive staining. **(D)** Vimentin staining with DAPI counterstain of LNCaP cells expressing GFP or Sox9. Insert shows higher magnification. **(E)** Western bot of LNCaP cells expressing RFP or SOX9 with antibodies against E-Cadherin, Vimentin and GAPDH. Quantitative RT-PCR of LNCaP cells expressing RFP or *Sox9* for **(F)** *Vimentin* and *CHD1* (*E-*Cahderin) and **(G)** *SNAIL*, *SLUG*, *TWIST* and *ZEB1*.

The highly invasive phenotype observed in Sox9;Pten;PBCre mutant prostates suggested an increase in epithelia to mesenchyme transition (EMT), which has been associated with SOX9 activity in other tissues. To address this possibility sections from Sox9;Pten;PBCre mutant prostates were stained with antibodies to E-cadherin or Vimentin together with GFP, to mark the cells expressing transgenic *Sox9*. Cells undergoing EMT have been shown to downregulate the expression of the epithelial marker E-cadherin and upregulate the mesenchymal marker vimentin. Our studies show that some GFP positive cells do show this phenotype and are particularly abundant in regions of high invasion (Figure 4A and 4B). Several key regulators of EMT have been shown to promote the loss of epithelial characteristics and the gain of mesenchymal traits [16]. Analysis of several of these regulators showed that there is an increase in SLUG (encoded by *Snai2*) and ZEB1 positive cells in Sox9;Pten;PBCre mutant prostates (Figure 4C). To investigate whether EMT is a direct effect of increased *Sox9* in prostate cells, we analysed this process in LNCaP cells that had high levels of *SOX9*. These cells had an increase in Vimentin positive cells when compared to control cells, however, E-cadherin was not found to be reduced (Figure 4D, 4E and 4F). Analysis of the expression of EMT regulators revealed an increase in *ZEB1* but not *SNAIL, SLUG* or *TWIST* in LNCaP cells with high SOX9 (Figure 4G) suggesting SOX9 can promote mesenchymal features in prostate cells, possibly through ZEB1.

### Cell fate identity of invasive cells in Sox9;Pten;PBCre mutant prostates

**Figure 5:**
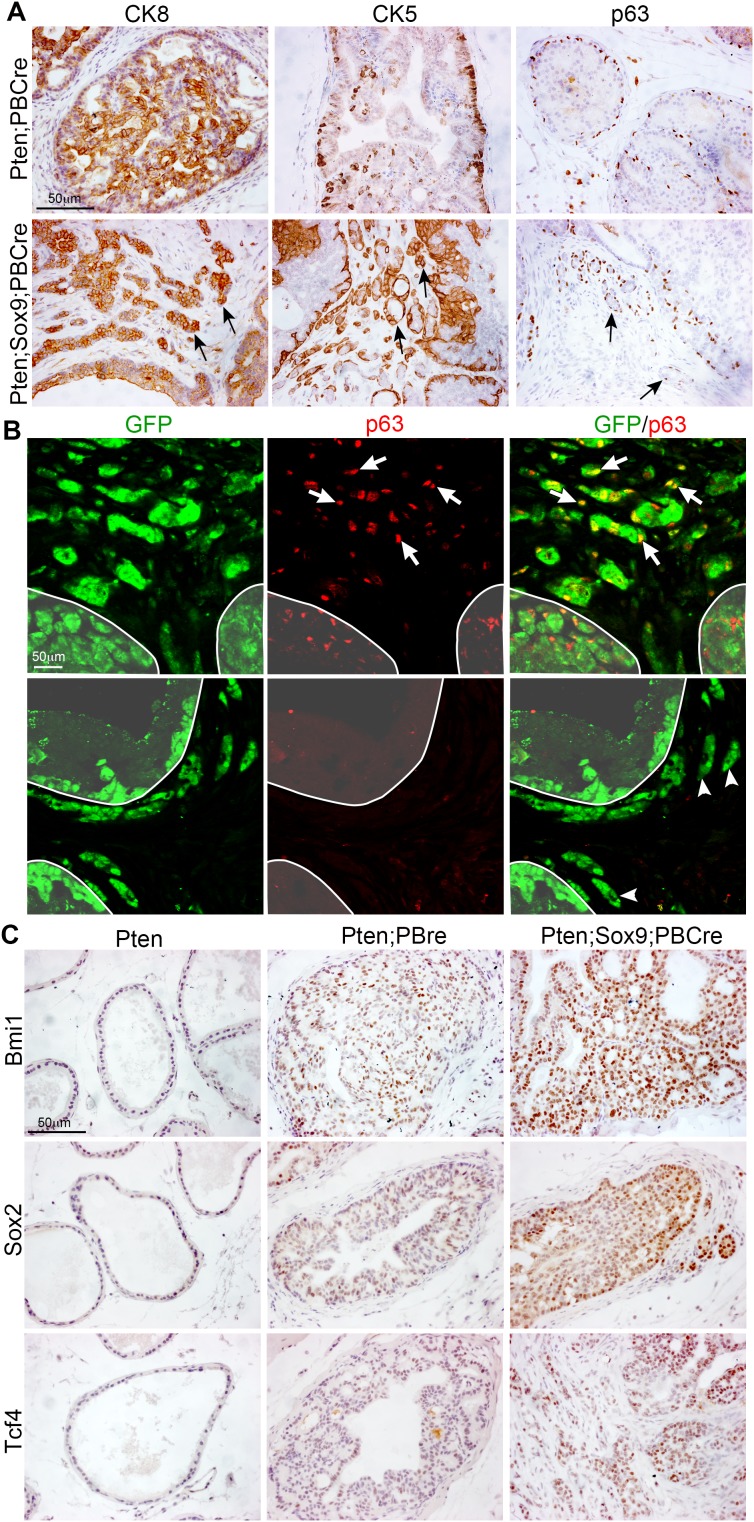
Sox9 promotes an increase in the expression of stem cell markers in *Pten* mutant prostate cells **(A)** CK8, CK5 and p63 staining of sections of prostates with *Pten* deletion or *Pten* deletion and *Sox9* overexpression. Arrows mark cells invading the stroma. **(B)** p63 and GFP immunofluorescence staining of sections of prostates with *Pten* deletion and *Sox9* overexpression. White arrows mark p63 and GFP double positive cells invading the stroma. White arrowheads mark p63 negative and GFP positive cells invading the stroma. White line demarcates the epithelial ducts. **(C)** BMI1, SOX2 and TCF4 staining of sections of *Pten* prostates, prostates with *Pten* deletion or prostates with *Pten* deletion and *Sox9* overexpression.

Prostate epithelial ducts are formed of basal and luminal cells. We therefore used antibody staining to investigate the cell fate identity of the invasive cells in Sox9;Pten;PBCre mutant prostates. Our studies revealed that invasive cells within the stroma express both luminal (CK8) and basal (CK5 and p63) markers (Figure 5A). Co-staining of Sox9;Pten;PBCre mutant prostates with GFP showed that some invasive regions had a high number of basal cells that coexpressed transgenic *Sox9* while others regions lacked basal cells (Figure 5B). The prostate basal cell population has been proposed to contain a high number of progenitor/stem cells [17, 18]. The relative increase in basal markers in Sox9;Pten;PBCre mutant prostates and the association of SOX9 with stem cell activity in other tissues, prompted us to look at stem cell markers. Antibody staining revealed an increase in BMI1, SOX2 and TCF4 in neoplastic regions of Sox9;Pten;PBCre prostates, including the invasive regions, relative to Pten;PBCre mutants (Figure 5C). This result was confirmed in LNCaP cells expressing high levels of *Sox9* that showed an increase in BMI1, SOX2 and TCF4 proteins (Supplementary Figure 3), although we could not detect p63 in these cells.

### Effect of castration on Sox9;Pten;PBCre mutant prostates

**Figure 6:**
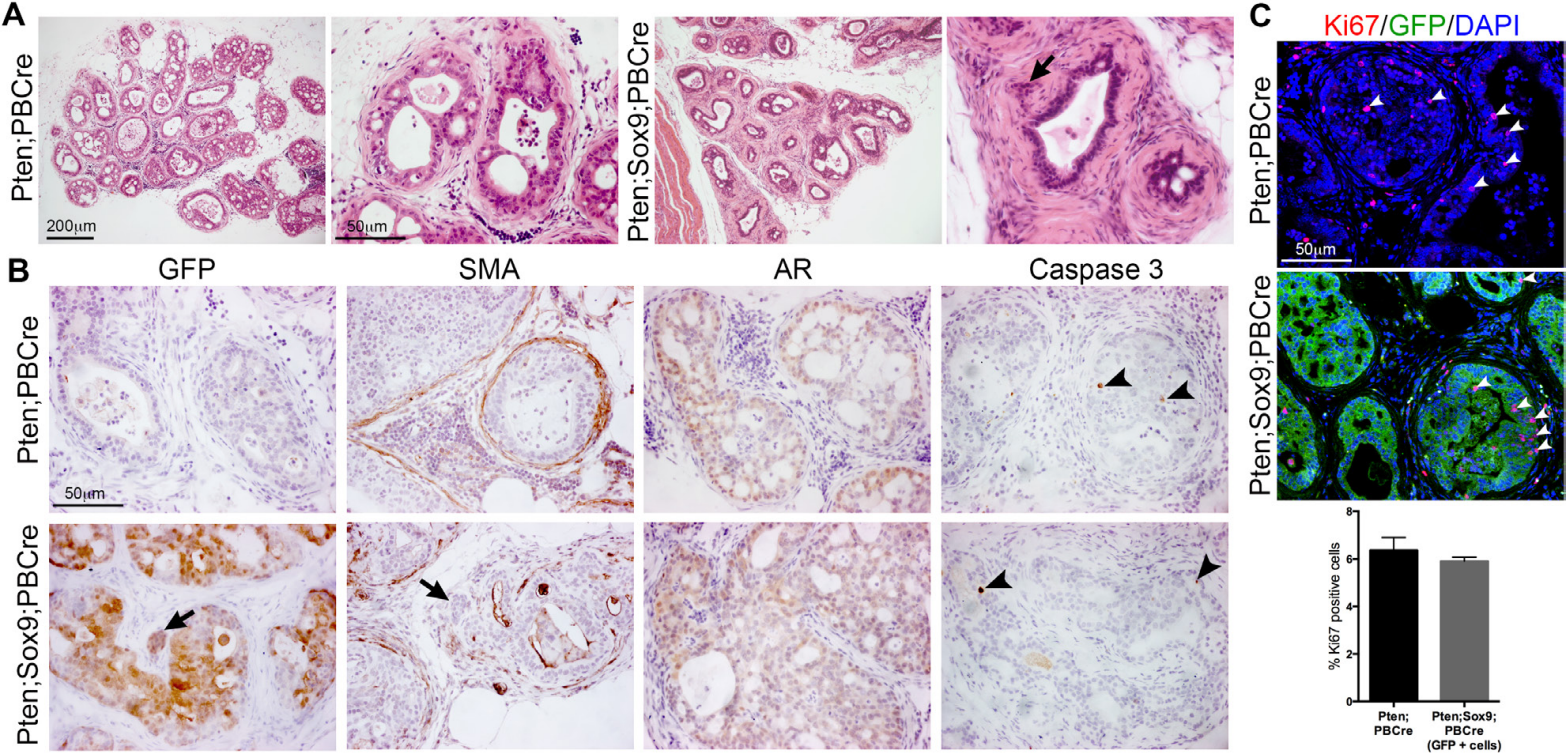
Castration of animals with *Pten* deletion and high *Sox9* does not prevent epithelial invasion into the stroma **(A)** Haematoxylin and eosin (H&E) staining of sections of prostates with *Pten* deletion or *Pten* deletion and *Sox9* overexpression from 3 month old animals castrated at 2 months. **(B)** GFP, smooth-muscle actin (SMA), Androgen receptor (AR) and Caspase 3 staining of sections of prostates with *Pten* deletion or *Pten* deletion and *Sox9* overexpression from castrated animals. Arrows indicate invading cells. Arrowheads indicate Caspase 3 positive cells. **(C)** Ki67 and GFP staining of sections of prostates with *Pten* deletion or *Pten* deletion and *Sox9* overexpression from castrated animals. DAPI is nuclear counterstain. White arrowheads indicate Ki67 positive cells. Bar chart showing quantification of Ki67 positive cells in prostates with *Pten* deletion or *Pten* deletion and *Sox9* overexpression from castrated animals.

Castration is the first line of therapy for prostate cancer patients in the clinic and SOX9 has been proposed to interact with AR [19]. Therefore we wanted to investigate whether the phenotype observed in Sox9;Pten;PBCre mutants was dependent on AR levels. Analysis of castrated mice showed that Sox9;Pten;PBCre mutant prostates were similar in size to Pten;PBCre mutant prostates, which were both smaller than their intact counterparts. However, the invasive phenotype was still observed in the Sox9;Pten;PBCre mutant prostate highlighted by the lack of stromal SMA staining surrounding the ducts (Figure 6A and 6B). Consistent with the lack of size difference, the levels of castration resistant proliferation, as measured by Ki67 staining, and apoptosis, as measured by Caspase 3 staining, were found to be similar between Sox9;Pten;PBCre and Pten;PBCre mutants (Figure 6B and 6C). Levels of nuclear AR staining were low in both mutants, a phenotype that was found in Sox9;Pten;PBCre mutant prostates that had not been castrated (Figure 6B and Supplementary Figure 4).

## DISCUSSION

SOX9 has been implicated in cancer in many tissues and its role has been shown to be varied and context dependent. We have been investigating the function of this factor in the normal and neoplastic prostate. Our previous work had shown that *Sox9* can promote tumour initiation in mice with a heterozygous *Pten* mutation. This study identifies a novel role for SOX9 as a driver of aggressive late stage invasive prostate cancer in homozygous *Pten* mutant animals. In addition, we show that its mechanism of action is to regulate several key processes that together promote highly invasive disease. Our study reveals that high *Sox9* levels lead to *Pten* mutant prostate epithelial cells increasing proliferation and acquiring properties of basal stem cells; to the induction of EMT, the deposition of extracellular matrix and changes in cytoskeleton and adhesion, properties that promote a highly invasive phenotype. Our analysis is consistent with SOX9 being a key regulator of these processes rather than regulating one event that brings about these phenotypes. A similar role has recently been proposed for this factor in basal cell carcinoma (BCC) [20].

Lineage plasticity has been proposed to be a mechanism not only of tumour progression to aggressive stages but also resistance to therapy [15, 21]. This change in cell fate is thought to occur through the increase in cells with stem like properties [22]. Recent transcriptome analysis has identified an association between human basal cells and aggressive prostate disease [18]. Our studies reveal that *Sox9* can induce a more basal-like phenotype in *Pten* mutant prostates. In addition, we identified an increase in the stem cell markers Sox2, Tcf4 and Bmi1 in both human and mouse prostate cells overexpressing *SOX9*. Direct regulation of *Bmi1* expression by SOX9 has been shown in mouse embryonic fibroblasts and proposed to occur in colorectal cancer [23]. A more indirect mechanism could give rise to the high levels of SOX2 in Sox9;Pten;PBCre mutants as loss of *RB* has been proposed to lead to an increase in *SOX2* expression, which was greatly augmented with concomitant *TP53* loss [15].

A striking effect of high levels of *SOX9* on LNCaP cells is their change in morphology and adhesion properties. In a study on BCC, *SOX9* has been shown to directly regulate genes involved in adhesion, cytoskeleton remodelling and extracellular matrix [20]. Consistent with this, we observe an increase in genes involved in cell shape and adhesion. Our work indicates that through the regulation of cytoskeleton dynamics and adhesion, SOX9 contributes to *Pten* mutant cells altering their epithelial properties and becoming more motile. The actin remodelling genes MACF1 and PALLD that we show are increased in SOX9 overexpressing cells have been shown to promote cell migration [24, 25]. Antibody staining on Sox9;Pten;PBCre mutant prostates revealed groups of invasive cells, suggesting a mechanism of collective cell migration. Presumably this process was further influenced by the ECM changes in the stroma.

SOX9 has been associated with the process of EMT in many tissues including embryonic neural crest [26]. Evidence of this process was found in Sox9;Pten;PBCre mutant prostates with a subset of GFP positive cells showing Vimentin staining. Increased staining was also observed in LNCaP cells expressing increased *SOX9*, although not in all cells. Moreover, an increase in *ZEB1*, an EMT regulator, was observed in both models. EMT is thought to be a transient process, therefore the sporadic staining we observed could reflect cells at different stages of the pathway.

Consistent with the highly invasive phenotype observed in Sox9;Pten;PBCre mutant prostates, we did find evidence of metastasis in tissues such as lymph nodes and lungs. However, in most cases these are single or small cell clusters suggesting that high levels of *SOX9* promote cell migration to secondary sites but other factors are required to establish colonisation and growth at these locations. A similar effect was observed in a breast cancer study where overexpression of *Sox9* led to micrometastasis in the lung but macrometastases were only observed when cells had increased levels of *Slug* [27].

Our results show directly that increased levels of SOX9 are required to drive both tumour initiation and invasive advanced disease and that it does this in association with other oncogenic pathways such as loss of *Pten*. *SOX9* is expressed in the normal prostate and mechanisms to achieve increased levels in tumours have been proposed. These include regulation of *SOX9* expression by *ERG* [10], which is found in tumours with TMPRSS2:ERG fusions, and loss of *Zbtb7a*, which encodes a factor proposed to antagonize SOX9 function rather than expression [11]. This dependence on levels might explain the focal nature of the phenotype in Sox9;Pten;PBCre mutant animals as only some cells achieve the required amount of protein to drive aggressive disease.

In conclusion, we identified a novel role of SOX9 as a driver of invasive prostate cancer, a property of aggressive disease. Our study shows that SOX9 is a key regulator of various cellular and molecular processes that together act to drive tumour progression. The genetically modified mice in this study are an ideal model to study the process of cell invasion and disease progression in prostate cancer and identify biomarkers of this process.

## MATERIALS AND METHODS

### Mouse strains

The *PBCre* transgenic mice (*ARR2PBCre*) have been described previously [13]. Mice with the conditional allele of *Pten* were obtained from The Jackson Laboratory [28]. The Z/Sox9 transgenic mice were kindly provided by Kathryn Cheah [9]. These male animals were bred on a mixed genetic background. All mouse work was carried out in accordance with the Institute of Cancer Research guidelines and with the UK Animals (Scientific Procedures) Act 1986.

### Mouse prostate histology

Histological phenotype of samples was assessed on haematoxylin and eosin stained sections. Serial sections were then stained for immunohistochemical analysis. Histological assessment was based on published guidelines and assisted by a pathologist [29]. An unpaired t-test was performed to test if there is a significant difference between the wet weight of prostate tumours.

### Quantification of cell proliferation

Proliferation was quantified by immunofluorescent stain of sections with an antibody against Ki67. The number of proliferating cells was calculated by counting the number of nuclear Ki67 stained cells with and shown as a percentage of the total number of cells stained with nuclear DAPI. *Sox9;Pten* mutant tumours were also stained with an antibody against GFP, and the number of Ki67 single positive cells and Ki67/GFP double positive cells were counted. Cells from at least 4 high power fields were counted per animal, which totalled more than 900 cells per animal. Three animals of each genotype were analysed. Randomly selected fields were counted for control analysis. An ANOVA or t-test was used to test if there was a significance difference in the number of proliferating cells between each group.

### Immunohistochemistry, immunofluorescence and western blotting

Mouse tissues were fixed overnight in 4% paraformaldehyde (PFA), dehydrated in an ethanol gradient series, washed in Histoclear and embedded in wax. Antigen retrieval was obtained by boiling sections in citrate buffer (0.1 M sodium citrate pH6 and 0.05% Tween) and sections were treated with 3% H_2_O_2_ to block endogenous peroxidase activity. Sections were blocked in 10% sheep serum and then incubated with primary antibodies in 1% sheep serum overnight. For DAB chromogen (Agilent) staining the ABC vector kit (Vector Laboratories) was used with biotinlyated secondary antibodies according to manufacturer's instructions and were counterstained with haematoxylin. Masson’s stain was performed using the Trichrome light green stain from TCS Biosciences (HS773-LG). For all mouse tumour stains, sections were processed from at least three animals of each genotype. For immunofluorescence cell staining, LNCaP cells were plated on coverslips, fixed for 20 minutes in 4% PFA and washed in PBS containing 0.01% Triton. Cells were then blocked in 10% sheep serum and incubated with primary antibodies in 1% sheep serum overnight. DAPI was used as a nuclear counterstain. TRITC labelled phalloidin (Thermo Fisher Scientific) was used to stain F-actin. Secondary fluorescent antibodies were obtained from Molecular Probes and were used at a 1:1000 dilution. Fluorescent images were visualized and collected on a Leica TCS-SP2 confocal microscope. Western blotting was performed using standard protocols, with cells lysed in RIPA buffer. The following antibodies were used; SOX9 (Millipore AB5535), GFP (Abcam ab13970), Ki67 (clone SP6, Abcam ab16667), Cyclin D1 (clone A-12, Santa Cruz sc-8396), p53 (Leica NCL-p53-CM5), p27 (Dako M7203), p16 (Leica NCL-p16-432), p19 (Abcam ab80), RB (clone EPR17512, Abcam ab181616), Smooth-muscle Actin (clone 1A4, Sigma A2547), Fibronectin (Agilent A0245), E-Cadherin (clone 36, BD Biosciences 610181), Vimentin (clone EPR3776, Abcam ab92547), ZEB1 (clone 3G6, Abcam ab180905), Slug (clone C19G7, Cell Signaling Technology 9585), CK8 (Covance MMS-162P), CK5 (Covance PRB-160P), p63 (clone 4A4, Santa Cruz sc-8431), BMI1 (clone D20B7, Cell Signaling Technology 6964), TCF-4 (clone 6H5-3, Millipore 05-511), SOX2 (Abcam ab97959), AR (clone PG-21, Millipore 06-680), pAKT (Ser473) (Cell Signaling Technology 9271), GAPDH (Abcam ab9485).

### Cell line work and lentivirus production and infection

LNCaP and 293T cells were STR profiled to confirm their identity. Both cell lines were maintained in RPMI1640 with 10% FCS and used at low passage number (less than 20).

*Sox9* overexpression was achieved by infecting cells with Lentiviral particles generated from the construct pLenti-GIII-CMV-SOX9-GFP-2A-Puro (LV318630, Applied Biological Materials Inc) and control cells infected with Lentivirus particles from the plasmid pLenti-GIII-CMV-RFP-2A-Puro (Applied Biological Materials Inc) or GIPZ-GFP (GE Dharmacon). Lentivirus particles were made in 293T cells by transfecting the Lentiviral plasmid with Lipofectamine 3000 (Thermo Fisher Scientific), together with the packaging plasmids psPAX2 and pMD2.G. Viral supernatants were collected, filtered, supplemented with 8 μg/ml polybrene and used to infect LNCaP cells.

### Cell adhesion assay

96-well plates were coated with 10 μg/ml Fibronectin (Sigma, F1141), 10 μg/ml Collagen I (BD Biosciences, 354236) or 10 mg/ml heat denatured BSA for 2 hours at room temperature and then washed. LNCaP cells were dissociating in TrypLE (Thermo Fisher Scientific) and seeded at 10,000 cells per well in 100 μl of media. Cells were incubated at 37°C for 1 hour, washed three times with PBS and fixed with 4% PFA for 20 minutes at room temperature. To quantify adhered cells, cells were stained with DAPI and the total number of cells per well were counted using the Celigo Imaging Cytometer (Nexcelom) with the Direct Cell Counting analysis.

### RNA isolation and quantitative RT-PCR

RNA was purified from cells using the RNeasy kit (Qiagen) and cDNA was made using SuperScript IV reverse transcriptase (Thermo Fisher Scientific), following the manufacturer’s instructions. Quantitative RT-PCR was carried out using Taqman gene expression assays using the following probes; *GAPDH* Hs03929097_g1, *CDH1* Hs01013958_m1, *VIM* Hs00958111_m1, *SNAI1* Hs00195591_m1, *SNAI2* Hs00161904_m1, *TWIST1* Hs00361186_m1, *MACF1* Hs00201468_m1, *GSN* Hs00609272_m1, *PALLD* Hs00363101_m1.

## SUPPLEMENTARY MATERIALS FIGURES


