# Supplementary material for: SOX9 is a driver of aggressive prostate cancer by promoting invasion, cell fate and cytoskeleton alterations and epithelial to mesenchymal transition
Source: Oncotarget. 2018 Jan 10;9(7):7604–15. doi: 10.18632/oncotarget.24123 (PMC5800928; doi:10.18632/oncotarget.24123)
Supplement: Supplementary file 1 [file oncotarget-09-7604-s001.pdf]

SOX9 is a driver of aggressive prostate cancer by promoting invasion, cell fate and cytoskeleton alterations and epithelial to mesenchymal transition

SUPPLEMENTARY MATERIALS

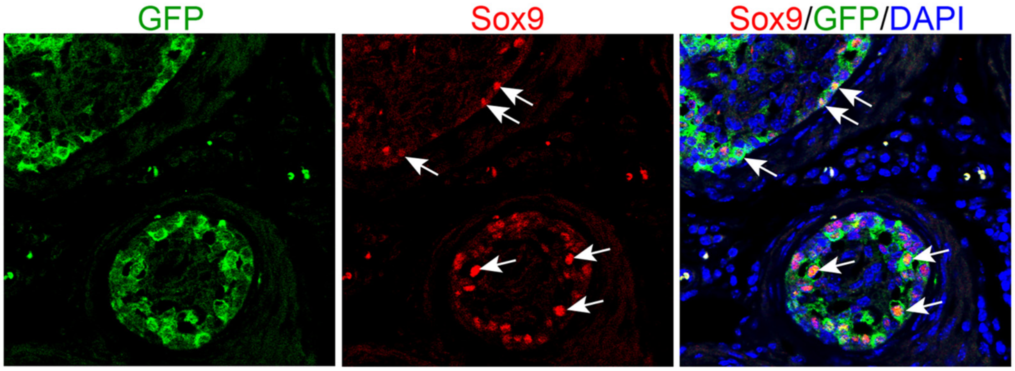

Supplementary Figure 1: GFP expression driven by the Z/Sox9 transgene correlates with high Sox9 expression in *Sox9;Pten;PBCre* prostates. GFP and Sox9 immunofluorescence staining of sections of prostates with *Pten* deletion and *Sox9* overexpression. Arrows mark cells that express GFP and have high levels of Sox9.

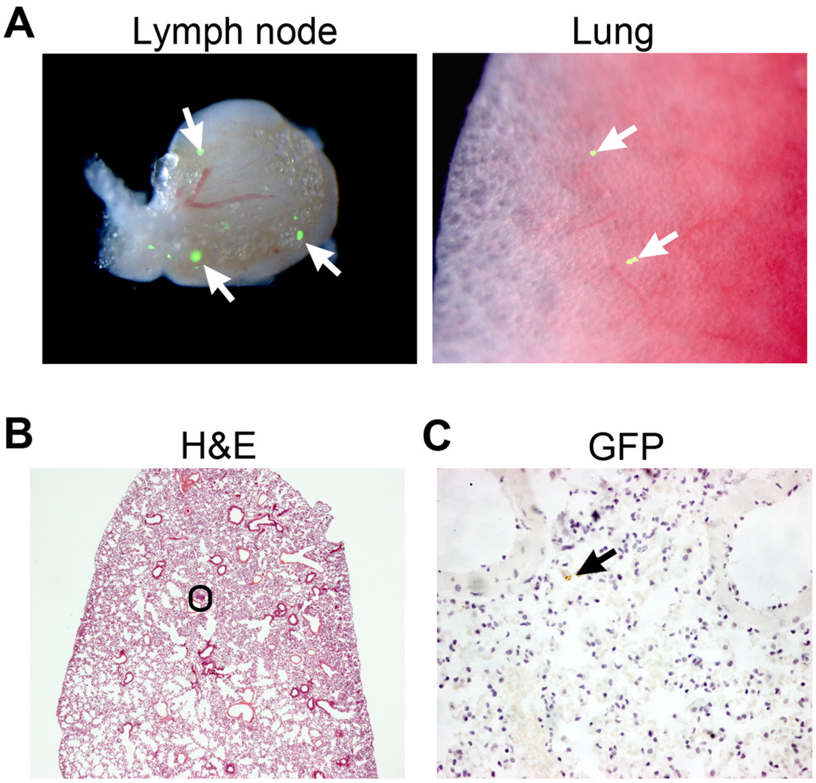

Supplementary Figure 2: Metastatic cells from animals with *Sox9;Pten;PBCre* mutant prostates. **(A)** Bright-field and overlaid GFP images of lymph node and lung from 6 month old animals with prostates that have *Pten* deletion and *Sox9* overexpression. White arrows indicate GFP positive metastatic cells. Haematoxylin and eosin (H&E) stain **(B)** and GFP stain **(C)** of lung sections from animals with prostates that have *Pten* deletion and *Sox9* overexpression. Circle highlights neoplastic cells. Arrow marks GFP positive cells.

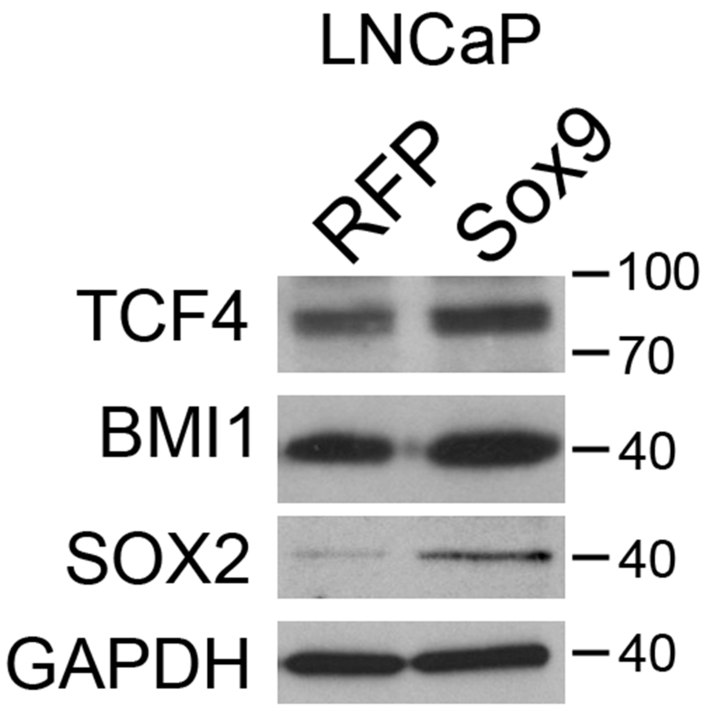

Supplementary Figure 3: LNCaP cells with high SOX9 have an increase in the expression of stem cell markers. Western blot of LNCaP cells expressing RFP or Sox9 with antibodies against TCF4, BMI1, SOX2 and GAPDH.

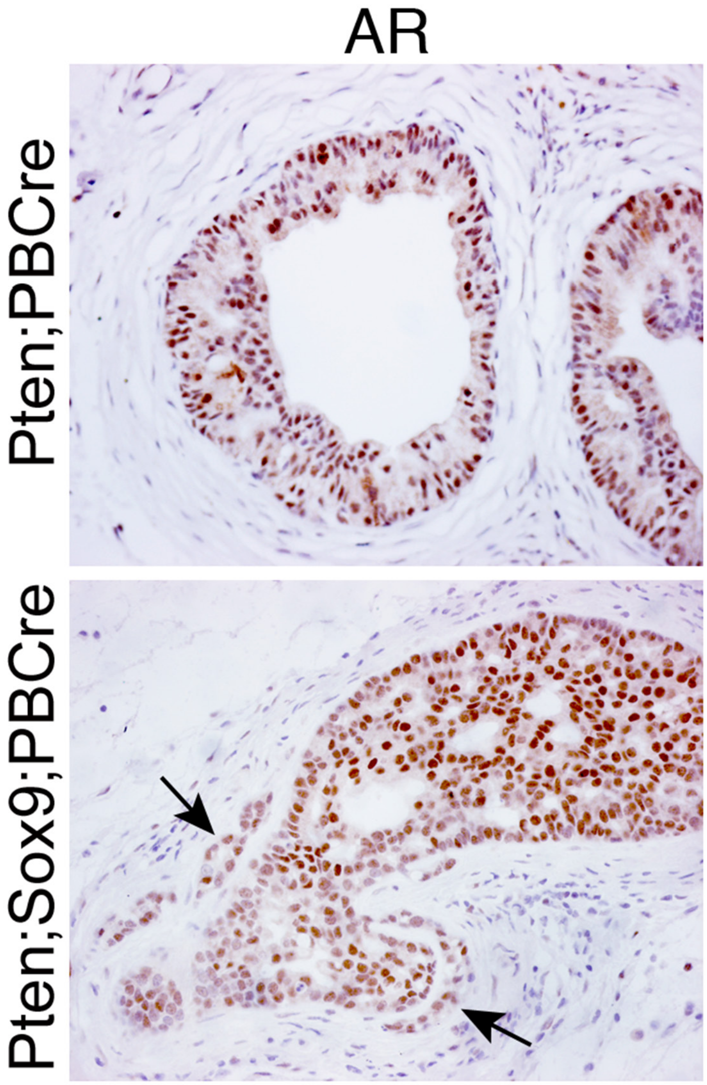

Supplementary Figure 4: Reduced AR expression in *Sox9;Pten;PBCre* mutant prostates. Androgen receptor (AR) staining of sections of prostates with *Pten* deletion or prostates with *Pten* deletion and *Sox9* overexpression. Arrows mark invading cells.
